# Not intervening as a form of care: Negotiating medical practices at the end‐of‐life

**DOI:** 10.1111/maq.12881

**Published:** 2024-08-27

**Authors:** Simon Cohn, Erica Borgstrom, Annelieke Driessen

**Affiliations:** ^1^ Department of Health Services Research and Policy London School of Hygiene and Tropical Medicine London UK; ^2^ School of Health, Wellbeing and Social Care The Open University Milton Keynes UK; ^3^ Department of Anthropology University of Amsterdam Amsterdam Netherlands

**Keywords:** end‐of‐life, intervention, palliative care, treatment

## Abstract

Biomedicine is organized around interventions. Despite growing concern about overtreatment in healthcare systems, not intervening can still raise questions about potential negligence and the quality of care. Based on ethnographic fieldwork with palliative care teams in England, we explore the work palliative care specialists do to reduce and sometimes halt interventions for patients at the end‐of‐life, in a general medical environment that is largely interventionist. We describe how judgments about what is an action or not aren't based on obvious or agreed criteria, but ultimately according to what different actors feel constitutes the best form of care. In other words, the underlying values that shape ideas of care determine how action and inaction are nominated, and not the other way around.

## INTRODUCTION

The story of Keith, a patient living with multiple sclerosis but now with limited time left, introduces a common feature of biomedicine; once set on a particular trajectory, clinicians are often committed to a cascade of options without really questioning their ultimate value:

When I saw the doctor, the first thing he said was, ‘Oh, we can do this, or we can do that…’ So I said, ‘No, you won't. You won't do any of those things, thank you very much.’ And then when he suggested a drug that will give me ‘an extra few months’, I replied ‘Does that give me an extra few months now, or an extra few months at the end? Because I want the few months now, I don't want them at the end.’

Here, Keith recounts how his doctor seemed compelled to suggest one treatment after another with the intention of prolonging his life, rather than acknowledge that because he was dying, a different approach might be more appropriate. His words serve to introduce our exploration into what happens when this medical impetus to always intervene encounters patients who will not get better and are formally acknowledged to be nearing the end of their lives. Drawing on ethnographic fieldwork conducted in London, United Kingdom, we focus on the work of palliative care staff, who often find themselves having to resist the wider imperative to always intervene in order to offer a different kind of care. We show how promoting alternative values and expectations often requires extensive negotiation with others which has the effect of differentiating palliative support from other areas of healthcare.

Many have previously described how biomedical practice is driven to always *do* something (Gawande, 2016; Good, 1993; Kaufman, 2015). This literature shows not only how the conflation between medical care and intervention is fostered from the very start of medical training (Atkinson, 1995) but also that the general emphasis on the so‐called “active treatment” is implicitly reproduced across diverse areas of healthcare, from outcome metrics (Crawford et al., 2011) to what counts as positive research findings (Chan et al., 2004). But just as crucially, this logic is also reproduced beyond the medical profession, in social expectations about what counts as “good” and “appropriate” care. Family members frequently share the assumption that the best medical care is always “active”—for example, in the form of further tests, new medication, or surgery (Neuberger et al., 2013). As a consequence, inaction or limits to intervening are regularly conceptualized as a failing (Bishop, 2011), while medical practice is often regarded as futile if no obvious curative options are available (Fox, 1980; Morciniec, 2020; Street & Kelly, 2021).

Recent debates about the tendency for health services to intervene unduly, coupled with a growing recognition of over‐diagnosis and societal over‐reliance on biomedicine, have galvanized the search for ways to reduce inappropriate or ineffective treatment (Kirkegaard et al., 2020; Ross et al., 2023). Such debates not only stem from the ever‐increasing burden put on healthcare resources but also from a growing recognition that overtreatment can actually be detrimental to health; for example, by exposing patients to unnecessary risks and side‐effects (Armstrong, 2018) or through the rise of antimicrobial resistance from excessive antibiotic usage (Tarrant & Krockow, 2022). However, much of this work tends to frame the problem in terms of safety or quality concerns, and finding direct, practical ways to change healthcare delivery (Heath, 2014; Lam et al., 2020). It therefore rarely addresses the entrenched assumption that “doing little” or “doing nothing” is, by default, equated with clinical error or medical neglect. As a result, this literature fails to recognize how intervening less, or sometimes not at all, is an equally important, deliberate and complementary facet of medical practice.

In this paper, we focus on end‐of‐life care in England, and explore everyday instances when medical staff decide not to do something, or when medications or interventions are decreased or halted (for more on our methodology, see Borgstrom et al., 2020). As we will show, accepting that death is likely in the near future serves to amplify and alter a range of expectations, hopes and values. But it is important to emphasize that we do not include instances of assisted dying or cases of euthanasia, both of which at the time of the research were not legal in the United Kingdom, because the intention driving what we describe is never to end a person's life. Nor should the instances we are interested in be regarded as examples of the so‐called “double effect,” in which an action to care for a dying patient may have the secondary effect of hastening their death (Tuckey & Slowther, 2009); in fact, the whole principle of the double‐effect is widely contested among palliative care professionals as being counter to their fundamental values (Hunt, 1998; Schwarz, 2004). So, while assisted dying and euthanasia are undeniably important topics which raise a wide range of cultural and legal issues that warrant anthropological scrutiny, we want to make a clear distinction between such instances when questions of life and death are linked to particular actions, and the more every‐day, small instances of crafting care through numerous deliberations and hesitations pertaining to whether to intervene or not. These latter features of palliative work get very little attention precisely because they do not represent significant, defining moments. We nonetheless argue that they are an integral characteristic of care.

Long‐term ethnographic fieldwork was conducted between 2018 and 2020 (before the start of the COVID pandemic), among two multidisciplinary palliative care teams based in London. While the term palliative care is frequently used synonymously with end‐of‐life care, strictly speaking it is a specialty designed to provide pain management and support for any patient with an incurable illness. Clearly this includes those people who are in the last months or years of their lives. But once a patient is deemed to be end‐of‐life, there are often additional concerns that palliative care addresses, including where someone may wish to die, specialized support for family members, and arrangements with other non‐clinical services. The specialty arose in response to critiques that death was being over‐medicalized and that the care being offered failed to consider the many psychosocial dimensions of dying (Clark, 2007). It is linked to the modern hospice movement, and while more established in high‐income countries like the United Kingdom, the United States, and Australia, palliative care is endorsed by the World Health Organization, with many initiatives to promote it globally (Clark, 2016; WHO, 2024; Zaman et al., 2017). In the United Kingdom, palliative care services have become embedded in both hospital and community settings and continue to espouse a so‐called “holistic approach” that extends beyond the typical biomedical remit (Seymour, 2012).

Despite the fact patients identified as being near the end of life know that they will not recover, there are many forms of intervention that can be conducted as part of palliative care; for example, starting a course of radiotherapy to slow down the progression of disease, or increasing doses of pain relief drugs. Just as significantly, decisions may also be made to reduce certain medications, halt procedures, or not begin a new intervention. It is for this reason that palliative care is frequently described as a move to “supportive” or “compassionate” care (Broom et al., 2013). It does not draw on different medical knowledge, but rather different values are mobilized; namely, with the acknowledgement that someone is dying, emphasis shifts from trying to cure them to making their last period of life as comfortable as possible.

Consequently, although not intended to be starkly distinguished from curative specialties, professionals working in palliative care often describe a tension, and sometimes an overt contradiction, with other areas of biomedicine. Our interest, consequently, is in how palliative care professionals not only resist the interventionist culture steered by the “logic of lifesaving” (Van Beinum, 2023), but find ways to communicate their approach to other medical colleagues, patients and family members. Indeed, against a general background in which other medics routinely talk to patients and their families about options and alternative strategies, the task to reframe intervening as not necessarily the best course of action is often challenging.

It should be noted that this option to not intervene is clearly very different from not being able to do anything because of external limitations, such as a scarcity of resources (for examples of such situations, see Livingston, 2012; Street, 2020). Instead, it arises when what matters most for someone can be pursued, performed and re‐established through specific care practices (Mol et al., 2010; Pols, 2006, 2023; see also the “What matters to you? initiative” e.g. O'Herlihy, 2018; End of Life Care Think Tank, 2021). However, even in the more recent care literature that foregrounds everyday practice, the notion of “tinkering”—referring to ongoing efforts to accomplish, temporarily, one “good” or another—invariably suggests acts of doing, rather than watchful waiting, hesitating, or refraining from action to seeing what happens. Here we consequently suggest that accounts of care and tinkering need to include acts of not doing as an integral part of—and not counter to—the concept of practice.

## OUR STUDY

A further dimension to this study stems from reflecting upon the epistemology of a great deal of research—the term itself referring to processes of seeking, and hence what gets counted as “findings.” In this vein, the social sciences have tended to focus on what is observed, said or done, presenting only these as data, rather than attending just as equally to what is not present or witnessed (Daston & Galison, 2007). As a result, only tangible activities and identifiable differences are taken note of and recorded as significant. The methodological challenge, therefore, concerns how to research things that are not done or not present (Frickel, 2014; Scott, 2018), and how to ensure that things that may not initially appear as “heuristically valuable” are nevertheless included (Brekhus, 1998; Thomas & Latimer, 2015). Additionally, because the categories of action and inaction are inherently relational, and can vary from different perspectives, the task is not simply to shift from the more obvious events and actions to focus on those things that are not done. So instead we sought out instances when inaction was raised *alongside* action, in order to preserve something of their contingent relationship.

Funding enabled us to conduct a long‐term ethnographic study of palliative care professionals and the patients and family members they supported.^1^ Unlike many other specialties in the healthcare system, palliative care spans community and acute settings, and includes clinicians, nurses, social workers, and allied health and social care professionals. Our project correspondingly worked with two overlapping multidisciplinary teams (one hospital based, the other community based). We were able to attend their regular meetings, follow individual staff over the course of their day, and observe the interactions they had with patients and relatives. Through these initial contacts, we also established longer term relationships with some of the patients and talked to them directly about their experiences. We encapsulate our overall approach as “shadowing” the service, repurposing a term regularly used in the medical profession to describe how a student or junior healthcare worker is typically expected to learn a particular role while being “on the job.” But for us, the symbolism of being present yet in the shadow of a staff member also resonated with our interest in the presence and character of absence, and between what is, and is not, overtly acknowledged.

The majority of data collection was conducted by Annelieke, while Simon and Erica remained in regular contact with the staff and participated in the various professional activities when they could. Ethical clearance was obtained by the university's ethics committee, as well as the standard ethics procedure in the UK National Health Service (NHS), plus additional necessary clearance because we potentially had access to confidential information.^2^ All the diverse elements arising from fieldwork—notes, transcripts, photographs and workshop outputs—were collated into a single data repository so that we could compare, link, and analyze everything together.^3^

To explore “not intervening” in palliative care practice, we take the categories of “doing” and “not doing” as linked ethnographic objects. We do not seek to define them in any absolute or philosophical way, but rather draw on our fieldwork to convey how they come to be divided and distinguished from each other by different groups of actors in specific situations. As we will show, the question of how and when to reduce or withdraw interventions is not only a major aspect of the work these healthcare professionals do; it is often interpreted as counter to approaches adopted by other clinical specialties which tend to pursue a cascade of interventions, even for those who are very ill and frail. Below, we foreground three different enactments of not doing at the end‐of‐life and describe how these inevitably emerge in relation to doing something: First, when doing everything becomes doing nothing; second, valuing not doing as “being‐with”; and third, when doing nothing gets reframed as doing something. In each, the work of palliative care staff includes helping others revaluate how “doing” is differentiated from “not doing,” resulting in a distinctive form of care being offered to those people who are dying.

## WHEN DOING EVERYTHING BECOMES DOING NOTHING

Patients identified as dying, and for whom all curative treatment options have been exhausted, are often the subjects of a change of direction, from doctors advocating that new tests or treatments should be attempted because they “have nothing to lose,” to the resigned stance that “nothing more can be done”.^4^ This latter stance marks a shift in the clinical agenda from “doing everything” to “doing nothing.” Because of this, many patients thereby have to cope with a double sense of finitude: not only that are likely to die in the near future, but that they may no longer receive treatment in the present.

It is usually at this juncture that medical staff may seek the help of the palliative care team, although this is not unproblematic. As one nurse summarized it, “I think some doctors take it as a bit of an insult that they have to get palliative care involved, like they've not been successful and haven't done their job well enough.” The underlying paradox to this statement is that while death may well be regarded as a kind of clinical failure, the vast majority of people in the United Kingdom—like most other high‐income countries—will nevertheless die while under direct medical care (Office for National Statistics, 2022). In contrast to many other areas of medicine, palliative care represents a specialist field that overtly champions a combination of intervening and not intervening in order to provide the most appropriate and meaningful form of care. Although not intended to be starkly separated from curative treatment, in practice, professionals working within this field regularly describe how their approach can lead to tensions with other areas of biomedicine.

Such a tension emerged around the case of Leo, an inpatient who was gravely ill with a type of blood cancer. When registrar Tanya visited him on the ward he was said to be receiving “active treatment”—a term commonly used to describe any medical intervention that has the potential to cure, even if that possibility is quite remote. Indeed, hematology is a specialty that is often referred to within the hospital as one that relentlessly pursues active treatment, so much so that the supplementary term “aggressive treatment” is sometimes used to acknowledge the fact interventions can come with quite dramatic risks and side‐effects. Perhaps this is because, when it comes to blood disorders, there is almost an endless number of new tests that can be conducted or different pharmacological cocktails that can be tried. Nevertheless, the palliative care team had eventually been informed about his case, as Leo's health continued to deteriorate.

While flipping through his medical notes, Tanya became visibly frustrated as she talked about a “lost window of opportunity” when Leo was still well enough to survive the transfer home, where he could have been cared for in coordination with social care and occupational health services. This had been his explicit wish, but because he had now stayed in hospital to undergo ever more tests and additional treatments, his health had become so precarious and fragile that the opportunity had passed. “I truly feel that we have failed him,” she lamented. Her concern was that by constantly striving to find an effective treatment, the hematology staff had unintentionally thwarted what mattered most to Leo. Their enactment of medical care—to keep trying to “do something”—prohibited what she felt was more appropriate; a form of care that would have allowed him to be looked after at home, ensuring he had a reasonable quality of life in his last days.

Although, in this instance, it was a specific hospital specialty that failed to shift away from the clinical imperative to try and cure, it is not just other medical colleagues who find it difficult to accept that a patient is dying and that therefore the kind of care has to alter. Palliative staff often have to manage patient and relative expectations as well, since they frequently share the same assumption that the best medical care is always to actively intervene. Carole was one such patient. We met her while she was waiting to see a surgeon about a tumor that had been detected in her head. Earlier, as a way of preparing her for bad news, her oncologist had suggested that future treatment could “just be palliative.” The word “just” was clearly the key signifier for Carole. She proudly reported how she had flatly told the doctor, “No, it bloody won't be.” She adamantly continued, “I'm not one for giving up and not trying.” So at this stage, although the palliative team had become involved, Carole's care straddled different specialties.

When Carole's surgeon finally arrived, he showed her an image on the screen—the tumor resembled a flat slug wrapping around the back of her brain. Looking for reassurance, Carole remarked that she was pleased it was not actually *in* her brain. The doctor acknowledged that while this was good, he was still very worried because it was so close to a major blood vessel. Tapping his pen on each of his fingers in turn, he listed a number of possible courses of action that could be followed, ranging from simply monitoring the tumor and administering pain relief to extensive surgery to try and remove as much of the cancer as possible. Carole immediately favored this last option, saying there was no way she wanted to leave the tumor inside her, and that she did not want to be a patient under the care of the palliative team. So, after further discussion, they jointly decided on a plan that entailed a number of interventions in addition to the operation. After surgery, she would have radiotherapy, possibly chemotherapy, and then ongoing steroid treatment. Just as Keith in the opening quotation described, there is often an unstoppable momentum once an interventionist logic has been settled upon, as one procedure inescapably follows another.

Even though the operation itself was successful and Carole was discharged from hospital after a few days, the radiotherapy—which entailed placing a large metal cage over her head—left severe burns on her scalp, while the chemotherapy made her continuously nauseous and constipated. These severe side‐effects increasingly postponed subsequent sessions, slowing down the overall treatment plan. Carole died at home a few months after her last round of radiotherapy—never managing to tolerate the entire course of interventions.

Together, Leo and Carole's stories indicate how, from an outsider's standpoint, what gets identified as an “active” intervention or treatment in everyday biomedical practice is perhaps not so straightforward. By categorizing certain medical practices as active, or even aggressive, others inevitably get characterized as ones of inaction and inertness, even though in reality they also require effort and expertise. The distinction is not based on the amount of time or degree of expertise involved, but rather on whether the care being provided is primarily directed to try and cure, or based on accepting a patient is dying and that the role of medicine needs to shift accordingly.

The approach of palliative staff therefore unsettles any simple tie between medical intervening and care. Although end‐of‐life patients have been formally identified as being in their last stages of life, there is rarely much sense of exactly when they will die. Dying therefore encompasses a great deal of uncertainty. This is reflected in the kind of care palliative staff give, in which the opposition between medically intervening or not, and indeed between life and death as absolute categories, get disrupted. Their professional outlook is consequently shaped by this sense of transition and liminality—not merely because of the progression of their dying patients, but also because their own role in the healthcare system is often seen to be situated between the customary medical approach of trying anything that may work, and providing no care at all.

## NOT DOING, BUT BEING WITH

Because palliative care staff are quite reflective about their role falling somewhat outside the more dominant medical imperative to always be demonstrably active, their concern is that this can become equated with feelings of futility and despair. The team often craft ways to address this. Some recounted how they occasionally invent things to do, even if they have little or no clinical benefit. For example, they may visit the bedside more frequently than necessary to check a patient's vital signs, simply because they want to be seen as actively caring even if recording this level of information is not clinically useful. They may inject inert solutions subcutaneously if family members express mistaken concern that their dying relative is not getting enough fluids. Or they may even support family members by suggesting they can help with small routine activities, such as providing water on a sponge to stop the patient's mouth becoming too dry or using a damp flannel to delicately wipe their brow or speaking to them even if they are unconscious. From one perspective, because care is so strongly associated with doing something, these are all instances when the palliative team feel compelled to find ways for them, and others, to be busy—even if those activities are thought to be clinically inconsequential. However, from another perspective, these are nevertheless genuine acts of care, even if they provide no clinical benefit. Care is inherently relational, and as much an enactment of the values of the giver as it is established by practical benefits for the receiver.

But in addition to these small acts of care, palliative staff also resist the overriding imperative to always be doing something through the idea that simply “being with” a patient has real value. The general manager of both palliative care teams, Hugh, who formerly worked as a nurse, talked about how sometimes just sitting next to a dying patient was the most valuable form of care staff could provide. He talked about the absence of typical medical action as actively allowing for a different kind of presence, which foregrounded patience, attentiveness, quietness, and connecting. This form of care was “more than just the physical.” He went on:

‘Being with’ may involve words; it may not. It may involve touch; it may not. But it is, you know, almost that walking side‐by‐side with somebody, through their particular journey. I think sometimes that is the hardest thing to do.

Here, Hugh poetically presents possibilities in pairs: speaking or silence; touching or not touching; doing or not doing. The effect is that the kind of care he conveys is purposely not attributable to either one or the other, but instead arises from keeping open the possibility of one thing in relation to its opposite. By shifting the focus to include different forms of not‐intervening alongside intervening, this account of care encompasses the many invisible, intangible, and quiet aspects that might normally get eclipsed. He ends his description by alluding to a final kind of pairing of staff members being “side‐by‐side” with patients, conveying a shared, interpersonal experience, characterized by a sense of continuity and ongoing presence.

Nina, a nurse specialist, was particularly passionate about the importance of sometimes simply spending time alongside a patient and resisting the compulsion to suggest new things to attempt. For her, a key role was to create this kind of supportive, peaceful space around the patient that protected them from the constant barrage of other people and their expectations. She likened this to siding with someone in the Colosseum who had to face the constant calls from onlookers to “keep fighting” and never give up. One of the senior doctors concurred, saying that just being with a dying person could nevertheless “make a difference,” even if nothing is done and no words are actually spoken. These apparently simple claims belie a very subtle reframing, not only of care, but also of the nature of their own agency and that of the patient. Spending time with someone, despite not actively intervening, is nonetheless a kind of active practice, because sometimes the most appropriate endeavor is to modestly shield a vulnerable patient from the bombardment of others.

Although palliative staff all share this commitment to simply being present in order to acknowledge the experiences of a patient, they do not necessarily find this easy to accomplish. Spending time without being obviously active makes them reflect on their professional role and the limitations of what they can do. “We are trained to *do* things,” one said. As they explained, the emotional intensity of being with a patient who is dying can make even the most experienced staff member feel uncomfortable. But being part of a team helps to mitigate this. Regular meetings, during which staff review their workload and discuss difficult, complex cases, also provide an opportunity to voice these experiences to colleagues, and through discussion, to reassert a sense of purpose.

The meetings begin as quite structured events. Lists of patients currently under the care of the team are read out, along with the names of those that have died over the last week. As these individual cases are then discussed, the recording of specific information follows a standardized checklist. But, as one senior member put it, “These are just the bare facts that clinical management can capture…. Most of what we do can never put into a form.” The reality is that when staff present individual cases, they often give extended, nonlinear narratives, even when there is no decision that needs to be made, or medical intervention that needs to be recorded. Weaving in details of apparently irrelevant things, such as a particular pet dog or a granddaughter's tears, the clinical task of differentiating what is relevant from what is not is circumvented. Instead, the description of a patient's entire circumstances interlaces medical considerations with a diverse range of other things. The effect is that the patient, as a unique person with an individual story, emerges from the account, and the role of “being with” gets recreated as a collective undertaking.

## REFRAMING NOTHING AS SOMETHING

As we have described, acknowledging that a patient is dying means that they, their relatives and the care service are often confronted with questions about whether to continue or halt a particular drug or other intervention, and hence make decisions about treatment that can be counter to usual clinical practice. But these kinds of consideration frequently extend beyond simply evaluating what is deemed to be clinically best, to contemplate how they might affect the overall quality of life and wellbeing for someone in their last stages. The palliative care team regularly describe this intrinsically open‐ended remit as their “holistic approach,” which may well conflict with how the wider medical profession comes to decide what is important.

Such a divergence of opinion arose for palliative nurse Tina, who was caring for Jane, a patient in her 60s with metastatic renal cancer. Jane had been discharged from the hospital on the basis that the oncology team had tried everything they could, and that the best thing would be if she was cared for at home. Unsurprisingly, the implicit message Jane took from being discharged was that there was nothing left that could be done. Tina wanted to find a way for Jane to understand that actually, although she would no longer receive treatment for her cancer, that did not mean that she would be left alone and not be looked after.

Now at home, Jane was suffering from intense discomfort; as Tina recalled, “I lost track of the many places where she had pain.” As a result, Jane had been taking an anti‐inflammatory drug, which she felt really helped. However, one of the other hospital doctors instructed her to stop taking it, on the grounds that prolonged use would eventually cause kidney damage. For Tina and the others in palliative team, this seemed nonsensical. There was no sign Jane had any kidney damage, and the fact that she was dying surely meant the focus should be on her present state, rather than a future possibility that was unlikely to be relevant. Nevertheless, there was actually no clinical evidence that the drug *could* offer any benefit. This became a bit of a conundrum for Tina and the team. Although they wanted to back Jane's wishes, especially because the risk of side‐effects was, in this instance, immaterial, doing so would effectively endorse Jane's claim that it reduced her pain and thereby contradict their own medical knowledge. In the end, they supported Jane, despite being certain the drug offered nothing medically significant, because it was nevertheless clearly something for Jane.

When a patient is thought to have very limited time left, the palliative team focus on making them as comfortable as possible. But doing so can be a delicate process and often requires careful negotiation, to ensure everyone appreciates that at this stage, being comfortable is not an inferior or secondary option but the most important priority. This work with patients, relatives and other staff involves reframing what might be considered a subsidiary concern and of limited significance into being the most crucial focus of care. This delicate work was certainly a priority for Natalie, a woman in her 60s with terminal lung cancer. Natalie had been in hospital for 3 weeks. Despite this, she was on one of the noisy, bustling Respiratory Medicine wards because—according to staff—she refused to be on a calmer oncology ward, which she associated with dying. During an initial visit, Sophie, one of the palliative nurses, reported that because of this, Natalie was “not really on anybody's radar,” and that she had to endure the hectic routines and activities of the ward.

On a subsequent visit, Natalie was sitting up in bed, surrounded by water bottles, a hand‐held fan and a cardboard sick bowl. She announced that she felt drowsy and nauseous. Sophie sat by the bed and asked whether she was in any pain and suggested that a syringe driver to administer medication might help. Natalie replied that she'd rather talk it over with the family, and not make any decision while she felt so exhausted. “You're the boss,” Sophie responded as she got up, “…we will make sure they do less to you… [and] talk to nurses about less obs [observations] and less bloods [tests] at night.” The implication was that because of the ward Natalie was on, the nurses were simply treating her like all their other patients. But this level of attention was actually disrupting her sleep and proving counterproductive.

The next day Sophie visited again, but this time with a consultant, Caitlin, so they could talk things over with the family. Everyone sat in a small room, while Natalie remained in her bed on the ward. Her husband, Roy, said that he had received a call from somebody to say it would be good if he came to the hospital. There was a moment of silence, and then Caitlin told him that some conversations had already happened in his absence, but that it would be really helpful if he could say what he understood about his wife's situation. Roy replied that he knew she was deteriorating. “Do you expect there might be any treatment in the future?” he asked. Behind Roy's back, his daughter Rose shook her head ever so slightly, while Jay, his son, sat silently with his partner. “We think your wife is just not fit for any treatment at the moment,” Caitlin replied delicately. Roy pushed back, asking if there was a timeline to things. Caitlin shifted in her chair, but rather than saying anything, turned to Rose. Rose looked directly at her father, saying, “Well, dad, we have talked about how mum is not doing well, and that it does not look so good. It may be weeks, but it may just be days.” Roy took a deep breath and started to describe how his father had died soon after he received a phone call from the hospital. He said that although he wouldn't expect one, he wouldn't be surprised if he got a call like that soon.

Caitlin wanted to alter the focus a little. She raised the possibility that Natalie might actually be aware of how ill she was, and perhaps should participate in the discussion. Rose answered that the family wanted to leave it to their mother to initiate such a conversation, even though this might mean they would not have one at all. Jay, the son, then spoke for the first time. He described his mother as a very private person and said that he didn't want her to die on the ward. Although single rooms are scarce, Caitlin said she would try her best to arrange one. A brief exchange focused on whether Natalie would appreciate a visit from the hospital chaplain. “Anything else?” Caitlin then asked tentatively, after what had been a difficult discussion. No one spoke, and then Roy began to sob. Perhaps it was the silence that allowed him to. Rose embraced him clumsily across the seat rests that divided them, whispering, “It's ok, it's ok,” but by then he had already removed his arm from his face and was gesturing that his emotions were now under control.

At no stage had Sophie, Caitlin, or the other members of the palliative care team tried to circumvent or ignore the intense and heartrending emotions experienced by Roy and the rest of the family. Nor had they tried to placate the difficult atmosphere by suggesting things that they could perhaps do. Their task was not to pretend to be optimistic, but instead foster a space in which everyone could come to terms with the reality that Natalie was dying, and that the best way to express their love for her was not to resist what was inevitable. Ensuring a final period of calm and reduced medical activity became the most meaningful act; much like the fleeting silence which had caused Roy to momentarily express his feelings.

Natalie died 4 days later. Although she had eventually been moved to a side room, she had become quite agitated during her final hours, to such an extent her family felt her death had been “terrible.” Despite this, Rose and the others were undeniably grateful for the care Natalie had received; the family raised money for the palliative team at the funeral. Natalie's story serves to illustrate just how much work and time is necessary for a palliative care team to help others accept and adjust to the situation, rather than identify a new course of action or default to the idea that there may be new interventions to try. Instead, their care shifts from more obvious medical practices to talking and sometimes staying quiet. The task is not simpy about transmitting information but using conversation and listening to establish a shared acknowledgement of the situation, and incrementally reshape expectations and perspectives.

## DISCUSSION

One of the underlying concerns that catalyzed this study was the fact that within biomedicine and beyond, not intervening is usually only interpreted as clinical neglect, rather than as a valid option that may be the preferable course of action. Drawing on our ethnography of end‐of‐life care, when this issue becomes particularly overt, we have explored how, in practice, the distinction between doing and not doing gets made in specific circumstances and is mobilized differently by groups of people. This can lead to potential frictions that arise when people draw the line discordantly. More specifically, accepting that a patient is likely to die in the near future inevitably shifts the medical goal from trying to restore their health to reducing pain and discomfort in the present. And because palliative care teams are committed to ensuring a patient's remaining life is as meaningful and fulfilling as possible, their scope widens beyond the normal biomedical one. This shift in purpose represents a significant change in the values that shape the decisions and actions taken.

The accounts above illustrate how decisions about whether to intervene emerge from a range of multiple and sometimes competing priorities encountered by individuals and groups, and according to specific circumstances. Non‐interventions are frequently made invisible by virtue of a lack of a mechanism, and language, to ensure they are recognized, understood, and acknowledged. Doing what others may initially categorize as “nothing”—such as withdrawing treatment or choosing not to intervene—necessitates palliative care staff invest additional time and resources to guide patients, family members, and often their medical colleagues to reframe their hopes and expectations of medical intervention. Kirby et al. (2021) have explored how palliative care teams in Australia use family meetings to recalibrate hope as something that is collectively produced. Our study shows that similar recalibration occurs around not‐intervening. The palliative care team have to work hard to ensure patients and others appreciate that doing nothing may be the best course of action, reframing it as something appropriate and positive. This is not an easy task because the sense of being active—or of knowing someone else is—is a much more strident response to dealing with mortality than the more quiet, understated commitment to offering support that acknowledges fragility and finitude, and the value of simply “being with” someone to share something of their experience.

Perhaps counter‐intuitively, our observations suggest that judgments about what constitutes an action and what does not are not based on obvious or agreed criteria, but ultimately are driven by what is *felt* to constitute the best care. In a similar vein, others have noted that what makes a death “good” or “bad” cannot be reduced to defined criteria, but is sensitively shaped by what options are possible for a particular patient (Buchbinder, 2018, 2021; Hurn & Badman‐King, 2019). We have extended this idea, by showing how conceptualizations of best care determine how action and inaction come to be nominated, and not the other way around. In this way, different specialist teams execute different *forms* of care which are characterized as much by contrasting values and goals, as by technical expertise or specialist skills. Thus, although some research suggests that palliative care and other specialties can function in parallel without fragmenting the overall delivery of care (see for example, Macartney et al., 2017), our study has emphasized just how much work palliative staff have to do in order to work with, and sometimes counter to, the interventionist ethos of other medical teams.

In this way, our study contributes to a growing body of literature on medical care, and more specifically how the end of life is made and understood in medical contexts. For example, Kaufman (2006) illustrates how medical interventionist logics and hospital structures in the United States often emphasize the prolonging of life. Similarly, in Buchbinder's study of abortion and medically assisted death, she describes how the legal framework and overall biopolitical infrastructure of North America inevitably encourage an interventionist approach that can undermine the agency of individual physicians (Buchbinder, 2022). We complement this work by focusing not on decisions that may have direct life or death ramifications, but instead the apparently less significant everyday practices of palliative teams, who craft a particular form of medical care by openly combining intervening with not intervening.

Nevertheless, we have also tried to make clear that this almost certainly is not the only area of medicine where care combines both decisions to act alongside decisions not to. For example, similar issues have been described by Bogicevic and Svendsen (2021) in their account of malleable decisions to enable patients to be part of oncology clinical trials. Indeed, it is almost certainly a common characteristic of all medical care. Complex multifaceted practices are not only a combination of different actions, but also pauses and omissions that can be just as active and significant. What seems key is the extent to which this is fully acknowledged and regarded as equally important. This article is therefore intended to be a case study of a far more general set of issues that not only could lead to productive comparative work in the future, but also prompt a reevaluation of what exactly we mean by a medical “practice” and the composition of medical care.

Finally, while most choices of terminology used by people in our study appeared to reproduce the logic that “not doing” was the opposite of “doing,” the cases themselves show how in real‐life contexts, acts of intervening and not intervening co‐constitute each other, and how the division is an expression of an underlying set of values. We therefore argue not only that there is value in highlighting the subtle ways in which different forms of care coexist within a hospital or community health service, but also that it might prove productive to adopt a different language that avoids discrete and oppositional thinking between doing and not doing. In turn, this might contribute to a wider shift of discourse, away from medicine's main purpose being to protect life and forestall death, to one in which its role is to continually engage with qualities of living, such that dying is no longer conceptualized as medicine's failure but as a vantage point from which to acknowledge the changing biological and social conditions everyone will experience.

## ENDNOTES

1 Grant reference: UKRI ES/P002781/1.

2 Formal ethical approval details: NHS HRA approval REC reference 18/LO/0871, IRAS number 239197.

3 Interviews with staff members conducted within the Forms of Care: Ethnographic Interviews about Palliative Care with Health Professionals in London, 2017–2021 are archived in a repository that can be accessed at UK Data Service, Colchester, Essex, United Kingdom. https://doi.org/10.5255/UKDA‐SN‐855055/

4 Our animation movie illustrates this phenomenon: https://www.open.edu/openlearn/endoflife/
